# Identification of THSD7B and PRMT9 mutations as risk factors for familial lung adenocarcinoma: A case report

**DOI:** 10.1097/MD.0000000000032872

**Published:** 2023-02-10

**Authors:** Qianqian Zhang, Yanwei Zhao, Zhaona Song, Qiang Zhang, Conghui Tian, Rongrong Li, Juan Zheng, Lili Yan, Mingliang Gu, Xiaodong Jia, Mingjun Li

**Affiliations:** a Department of Joint Laboratory for Translational Medicine Research, Liaocheng People’s Hospital, Liaocheng, China; b Department of Radiotherapy, Liaocheng People’s Hospital, Liaocheng, China.

**Keywords:** familial LUAD, PRMT9, THSD7, WES

## Abstract

Lung tumors arise from the unrestrained malignant growth of pulmonary epithelial cells. Lung cancer cases include both small and non-small cell lung cancers, with lung adenocarcinoma (LUAD) accounting for roughly half of all non-small cell lung cancer cases. Research focused on familial cancers suggests that approximately 8% of lung cancer cases are linked to genetic susceptibility or heritability. The precise genetic factors that underlie the onset of lung cancer, however, remain to be firmly established.

**Patient concerns:**

A 43-year-old presented with nodules in the lower left lung lobe. Following initial antibiotic treatment in a local hospital, these nodules remained present and the patient subsequently underwent the resection of the left lower lobe of the lung. The patient also had 4 family members with a history of LUAD.

**Diagnosis:**

Immunohistochemical staining results including cytokeratin 7 (+), TTF-1 (+), new aspartic proteinase A (+), CK5/6 (−), P63 (−), and Ki-67 (5%+) were consistent with a diagnosis of LUAD.

**Intervention:**

Whole exome sequencing analyses of 5 patients and 6 healthy family members were performed to explore potential mutations associated with familial LUAD.

**Outcomes:**

Whole exome sequencing was conducted, confirming that the proband and their 4 other family members with LUAD harbored heterozygous THSD7B (c.A4000G:p.S1334G) mutations and homozygous PRMT9 (c.G40T:p.G14C) mutations, as further confirmed via Sanger sequencing. These mutations were predicted to be deleterious using the SIFT, PolyPhen2, and MutationTaster algorithms. Protein structure analyses indicated that the mutation of the serine at amino acid position 1334 in THSD7B to a glycine would reduce the minimum free energy from 8.08 kcal/mol to 68.57 kcal/mol. The identified mutation in the PRMT9 mutation was not present in the predicted protein structure. I-Mutant2.0 predictions indicated that both of these mutations (THSD7B:p.S1334G and PRMT9: p.G14C) were predicted to reduce protein stability.

**Lessons:**

Heterozygous THSD7B (c.A4000G:p.S1334G) and the homozygous PRMT9 (c.G40T:p.G14C) mutations were found to be linked to LUAD incidence in the analyzed family. Early analyses of these genetic loci and timely genetic counseling may provide benefits and aid in the early diagnosis of familial LUAD.

## 1. Introduction

Lung tumors arise from the unrestrained growth of pulmonary epithelial cells. Among men, lung cancer remains the leading driver of cancer incidence and morbidity, while it is second only to breast cancer among women with respect to mortality according to GLOBOCAN 2021 statistics.^[1]^ Lung cancer cases are classified into small cell lung cancer (SCLC) cases and the more common non-SCLC (NSCLC) cases, which make up 80% of total lung cancer incidence and include lung adenocarcinoma (LUAD), squamous cell carcinoma, and large cell carcinoma cases.^[2]^

LUAD is the most frequently diagnosed histological subtype of NSCLC and accounts for roughly half of all lung cancer cases.^[3]^ A novel multidisciplinary approach to LUAD classification was proposed by the International Association for the Study of Lung Cancer, the American Thoracic Society, and the European Respiratory Society in 2011, classifying tumors into those exhibiting scaly, papillary, micropapillary, acinar, and solid growth based on microscopic examination of the tumor growth patterns.^[4]^ Pathological analyses remain the gold standard approach to LUAD diagnosis, and immunohistochemistry staining can offer an effective means of maximizing the diagnostic utility of relatively small tissue biopsy samples. IHC targets analyzed when diagnosing LUAD cases at present generally include thyroid transcription factor 1, new aspartic proteinase A, and cytokeratin 7.^[5]^ During the early stages of disease, LUAD generally exhibits an absence of any symptoms and as it progresses it often exhibits diverse complex manifestations such that instances of misdiagnosis and missed diagnosis are common, hampering efforts to effectively draw the attention of patients and their families to this deadly disease. Accordingly, most patients are only diagnosed in the middle or advanced stages when it is too late for surgical tumor resection. This, coupled with the general resistance of LUAD cases to chemotherapeutic treatment, makes the early diagnosis of affected patients particularly important.

The identification of key driver genes including EGFR, KRAS, BRAF, ALK, HER2, MET, RET, and ROS1 revolutionized the treatment of LUAD. EGFR is among the most common LUAD-related driver genes, and is mutated in an estimated 60% of all female Asian nonsmoker LUAD patients.^[6]^ While major advances in the early diagnosis, targeting, and immunotherapeutic treatment of this disease have been made in recent decades, overall LUAD patient survival rates remain poor, largely as a result of the limited current understanding of the mechanistic drivers of this form of cancer and the cellular pathways dysregulated within these tumors.^[7]^

Familial cancer is a term that refers to families in which multiple individuals are diagnosed with the same form of cancer, providing a valuable case series for efforts to better understand the mechanisms underlying specific cancer types.^[8]^ Studies of familial lung cancer have led to the estimate that roughly 8% of all lung cancer cases are heritable or linked to some form of genetic susceptibility. Owing to genetic recombination, first-degree relatives of lung cancer patients that are nonsmokers face a higher risk of developing lung tumors as compared to the general population.^[9–12]^ Even so, the precise genetic factors that contribute to lung tumor susceptibility are incompletely understood. Further research focused on familial lung cancer thus has the potential to provide new insight into the mechanisms that govern this disease, aiding in the identification of previously unrecognized oncogenes and tumor suppressor genes.

In the present study, whole exome sequencing (WES) was used to evaluate 5 patients and 6 healthy individuals from a family affected by familial LUAD in Liaocheng Shenxian. The in-depth analyses of these sequencing results were used to establish candidate genes related to the pathogenesis of familial LUAD as a means of better understanding germline factors related to LUAD incidence. Together, these analyses have the potential to define key molecular target that can be leveraged for genetic screening, diagnostic efforts, and treatment of individuals at risk for familial LUAD.

## 2. Case report

**Figure 1.**
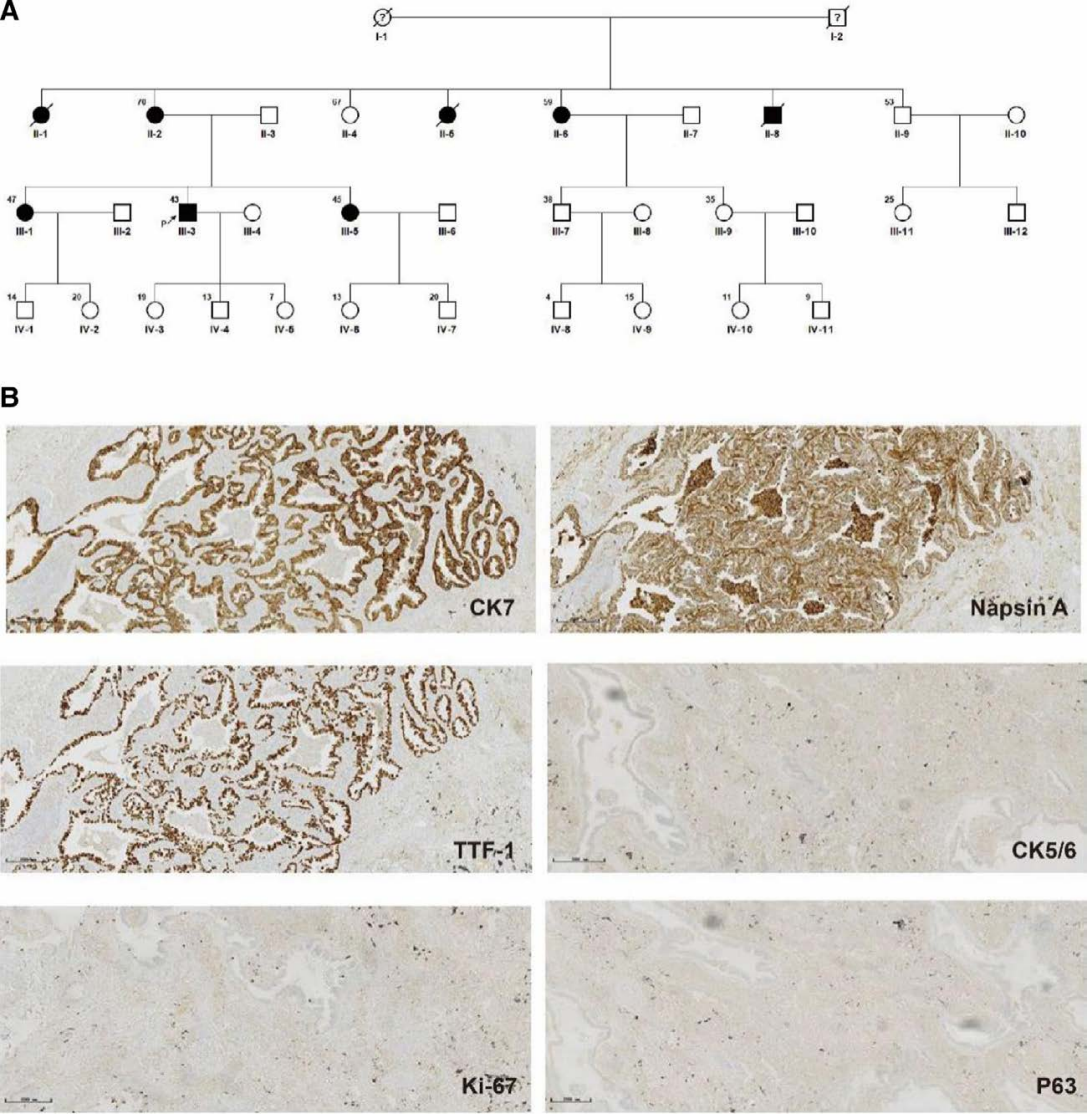
(A) Family pedigree. (B) Immunohistochemical staining of pathological sections from the proband: CK7 (+), TTF-1 (+), Napsin A (+), CK5/6 (−), P63 (−), and Ki-67 (5%+). CK7 = cytokeratin 7, Napsin A = new aspartic proteinase A.

In total, 35 members from this family affected by familial LUAD were evaluated, of whom 30 are still alive and 5 have died. Two individuals in generation I died of unknown causes, while 3 from generation II died of lung cancer (Fig. 1A). Samples were collected from eleven members of this family including LUAD patients II-2, II-6, III-1, III-3, and III-5 as well as healthy individuals II-3, II-4, II-9, III-9, IV-2, and IV-4. These samples were used for WES. The Ethical Review Committee of Liaocheng Hospital approved this study, and all participants provided informed consent prior to blood sample collection.

Proband III-3 (43 years old) presented with nodules in the lower left lobe of the lung, but did not exhibit any evidence of chest pain, chest tightness, wheezing, palpitations, intermittent coughing, sputum production, blood in the sputum, hoarseness, fever, dizziness, headache, abdominal pain, nausea, vomiting, or diarrhea. The patient exhibited good living and working environments and did not engage in risk-related habits such as smoking, alcohol intake, or drug use. The patient was initially treated with antibiotics in a local hospital, but this did not have any effect on the observed lung nodules. These lesions were subsequently resected. IHC staining results included cytokeratin 7 (+), TTF-1 (+), new aspartic proteinase A (+), CK5/6 (−), P63 (−), and Ki-67 (5%+), supporting a diagnosis of LUAD (Fig. 1B). Patients II-2, II-6, III-1, and III-5 were also diagnosed with LUAD based on pathological puncture results.

Data quality control was performed using Trimmomatic, with adaptor sequences and those that failed to meet quality control criteria being removed. Burrow-Wheeler Aligner was used to align sequences to the hg38 human genome, and single nucleotide polymorphisms (SNPs) and insertions/deletions were detected via GenomeAnalysisTK-3.8. ANNOVAR, snpEff, and CADD.SNVs were used to annotate identified variants, while high-risk InDels were filtered by identifying non-synonymous mutations in exons and splice site regions, mutations with a minimum allele frequency < 5%, and mutations with a snpEff prediction score > 15. Eight known driver genes (EGFR, ALK, ROS1, BRAF, MET, HER2, KRA, and RET) were screened for high-risk mutations.

The average sequencing depth achieved for the target capture region was 122x, with sequencing coverage of over 99.9%. The sequencing coverage was >99.9%. On average, 280,499 mutation sites were detected per sample. No high-risk mutations in any of the 8 analyzed genes were observed in the proband. However, further screening of high-risk mutation sites in these samples identified 2 high-risk SNPs: *THSD7B* rs371555754 (NM_001316349), which was present as a heterozygous mutation in patients but not in healthy individuals, and *PRMT9* rs78226695 (NM_138364), which was present as a homozygous mutation in patients but absent or present as a heterozygous mutation in healthy individuals (Table 1).

**Table 1:** SNPs exhibiting significantly different allele frequencies.

Chr	Gene	SNPs	Exon	Allele	AAChange.refGene	cytoBand
Chr2	THSD7B	rs371555754	22	A > G T: 0/1; N:0/0	c.A4000G:p.S1334G	2q22.1
chr4	PRMT9	rs78226695	1	G > T T:1/1; N: 0/0, 0/1	c.G40T:p.G14C	4q31.23

N = normal samples, SNP = single nucleotide polymorphism, T = tumor samples.

**Figure 2.**
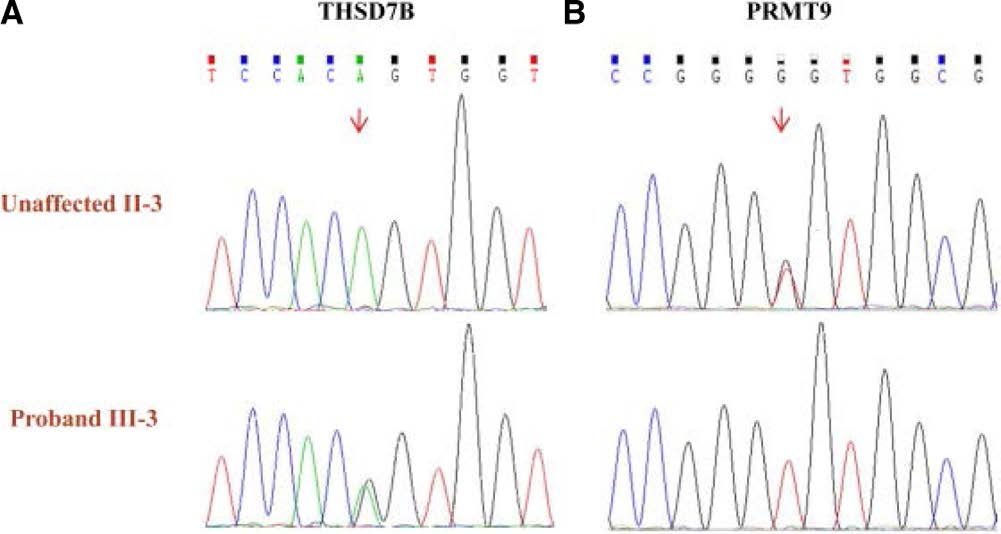
Sequencing peaks for candidate pathogenic genes. (A) A portion of the THSD7B sequence from unaffected individual II-3 and proband III-3, with mutation sites marked using arrows. (B) A portion of the PRMT9 sequence from unaffected individual II-3 and proband III-3, with mutation sites marked using arrows. Individuals II-3 and III-3 were heterozygous and homozygous for the identified mutation, respectively.

Sanger sequencing indicated that the proband III-3 and patients II-2, II-6, III-1, and III-5 in this family all carried these identified THSD7B and PRMT9 mutations (Fig. 2). In contrast, healthy individuals IV-1, IV-3, and IV-6 who did not exhibit any evidence of LUAD were heterozygous carriers for the THSD7B mutation. However, the absence of LUAD in these individuals may be related to a range of factors including age and environmental variables. Accordingly, these individuals should undergo routine physical examinations over time to monitor for any potential evidence of LUAD onset given the uncertain risk associated with their heterozygous carrier status.

**Figure 3.**
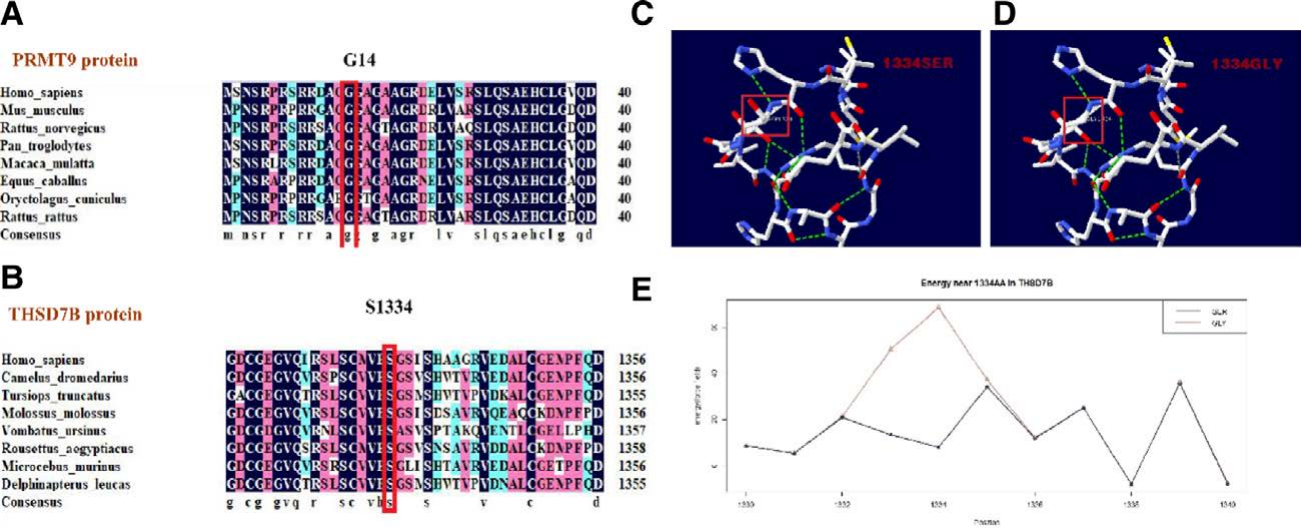
(A) PRMT9: Gly 14 (B) and THSD7B Ser 1334 across species. (C–E) Schematic diagrams of the 3D protein structure of THSD7B with prior to and after the C > D mutation, which altered the amino acid at position 1334 from a serine to a glycine. Changes in the force field proximal to this amino acid are shown. Red boxes indicate the amino acid at this site prior to and after mutation, while hydrogen bonds are represented by green dotted lines, black lines represent energy prior to this change in amino acid sequence, and red lines represent the energy after this change in amino acid sequence.

DNAMAN was next used for amino acid sequence homology comparisons, revealing that the PRMT9: 14Gly and THSD7B: 1334Ser residues were highly conserved across several species. Higher levels of conservation suggest that the mutation of a given residue is likely to have an even more pronounced adverse impact (Fig. 3A and B). Potential harmful effects of these mutations on protein function were examined using SIFT, Mutation Taster, and PolyPhen 2, revealing the PRMT9 c. 40 G > T mutation to be potentially damaging while the THSD7B c. 4000 A > G mutation was identified as being pathogenic (Table 2). Protein structural analyses indicated that the serine to glycine mutation at amino acid position 1334 in THSD7B reduced the minimum free energy for this protein from 8.08 kcal/mol to 68.57 kcal/mol (Fig. 3C–E). The identified PRMT9 mutation was not present in a region that directly altered protein structure. The I-Mutant2.0 results for both of these mutation sites suggested that THSD7B:p.S1334G and PRMT9: p.G14C would reduce the stability of their corresponding proteins (Table 2).

**Table 2:** Predicted SNP pathogenicity.

SNPs	SIFT	Polyphen2_HDIV	MutationTaster	Pro_Mut_Pos	I-Mutant2.0
rs371555754	Tolerated	Benign	Disease_causing	THSD7B (1334:S > G)	−3.27(<0)/Stability decrease
rs78226695	Deleterious	Possibly damaging	Polymorphism_automati	PRMT9 (14:G > C)	−0.29(<0)/Stability decrease

SNP = single nucleotide polymorphism.

## 3. Discussion

Lung cancers, which include both NSCLC and SCLC cases, remain among the most common and deadliest malignancies in the world.^[13]^ While the 2 greatest known risk factors for lung cancer incidence are environmental pollution and smoking, genetic factors can also shape the risk of lung cancer development. Here, a WES analysis of LUAD cases in a specific family in Liaocheng Shenxian was performed, leading to the identification of 2 SNPs associated with familial LUAD: *THSD7B* rs371555754 (NM_001316349) and *PRMT9* rs78226695 (NM_138364).

THSD7B encodes thromboresponder protein type 1 domain 7B, which is a membrane component that plays a role in actin cytoskeletal reorganization and is involved in the post-translational modification and glycosylation of proteins. Mutations in THSD7B have the potential to suppress cell death-related signaling activity while promoting the upregulation of invasion and metastasis-related pathways and downregulating immune response pathways, thereby impacting SCLC patient prognosis.^[14]^ The THSD7B rs13405020 SNP is also known to be related to prognostic outcomes in individuals with NSCLC.^[15]^ Protein interaction analyses have demonstrated that THSD7B is capable of interacting with both SPON family proteins as well as THBS1. SPON2 is associated with cellular adhesion,^[16]^ whereas THBS1 is involved in angiogenic and tumorigenic activity such that it is frequently mutated in the context of cancer progression.^[17]^ Heterozygous *THSD7B* variants can result in decreases in cellular adhesion together with enhanced tumor cell invasivity and metastasis.

The type II protein arginine methyltransferase encoded by the PRMT9 gene serves as a regulator of alternative splicing.^[18]^ PRMT9 upregulation has been reported in hepatocellular carcinoma tumors relative to healthy paracancerous tissue, with such overexpression being correlated with poorer tumor differentiation, vascular invasion, and more advanced Tumor Node Metastasis staging.^[19]^ In protein interaction analyses, PRMT9 was predicted to interact with both PRMT5 and SF3B2. PRMT5 is another known regulator of oncogenic development through its ability to regulate p53 pathway signaling and to drive the proliferative, invasive, and metastatic activity of tumor cells.^[20]^ SF3B2 has been linked to a range of cancers such as pancreatic adenocarcinoma, renal cell carcinoma, and papillary carcinoma. Homozygous PRMT9 mutations may result in abnormal transcript slicing, disordered transcriptional regulation, and consequent changes in cellular function that can result in pro-tumorigenic proliferation and migration.

In summary, mutations in the THSD7B and PRMT9 genes are associated with the incidence of LUAD in the analyzed family. Additional studies will be necessary to better understand the roles that these THSD7B and PRMT9 mutations play in the pathogenesis of familial LUAD in these individuals. Overall, these results offer novel insight into the genetic basis for familial LUAD while offering a foundation for future efforts to better study this form of heritable cancer rise and to diagnose susceptible individuals at an earlier stage of disease progression.

## Author contributions

**Conceptualization:** Qianqian Zhang, Mingjun Li.

**Data curation:** Qianqian Zhang, Zhaona Song.

**Formal analysis:** Rongrong Li.

**Funding acquisition:** Juan Zheng, Xiaodong Jia.

**Methodology:** Qiang Zhang, Conghui Tian, Lili Yan.

**Resources:** Yanwei Zhao.

**Software:** Zhaona Song.

**Supervision:** Qianqian Zhang.

**Writing – original draft:** Qianqian Zhang.

**Writing – review & editing:** Mingliang Gu.
